# Artesunate alleviates cisplatin-induced nephrotoxicity by regulating tryptophan metabolism and inhibiting ferroptosis

**DOI:** 10.3389/fphar.2026.1884026

**Published:** 2026-09-14

**Authors:** Haifeng Hu, Wenting Zhang, Huilong Wang, Shengpeng Zhang, Shaozhen Wang, Yuewu Xie

**Affiliations:** School of Pharmacy, Wannan Medical University, Wuhu, China

**Keywords:** artesunate, cisplatin, ferroptosis, nephrotoxicity, tryptophan metabolism

## Abstract

**Introduction:**

Cisplatin (CIS)-induced nephrotoxicity is a dose-limiting adverse effect that hinders its clinical efficacy. Artesunate (ART) exerts well-documented anti-inflammatory and anti-oxidative effects, but its potential efficacy against CIS-induced nephrotoxicity remains poorly elucidated.

**Methods:**

The renoprotective effects of ART were evaluated in a murine CIS-induced nephrotoxicity model and human HK-2 renal tubular epithelial cells. Renal function, histopathological injury, inflammation, and oxidative stress were evaluated. Untargeted renal metabolomics and transcriptomics were integrated to identify potential pathways, followed by targeted biochemical and molecular validation. Indoxyl sulfate rescue experiments were performed to investigate the functional relationship between tryptophan metabolism and ferroptosis.

**Results:**

ART treatment significantly ameliorated CIS-induced renal dysfunction, as evidenced by reduced SCr and BUN levels and attenuated histopathological damage. These functional improvements were accompanied by marked suppression of pro-inflammatory cytokines and restoration of redox balance. Integrated renal metabolomic and transcriptomic analyses revealed that ART exerts its protective effects primarily through modulation of tryptophan metabolism and ferroptosis pathway. Subsequent validation confirmed that ART inhibited ferroptosis by reducing Fe^2+^ accumulation, downregulating ACSL4 and FTL, and upregulating GPX4 and xCT. In HK-2 cells, indoxyl sulfate partially counteracted the cytoprotective and anti-ferroptotic effects of ART. Moreover, ART did not diminish CIS’s antineoplastic effect in human cancer cells.

**Conclusion:**

These findings demonstrate that ART alleviates CIS-induced nephrotoxicity by restoring tryptophan metabolic homeostasis and suppressing ferroptosis, positioning ART as a promising therapeutic candidate for preventing nephrotoxicity in patients undergoing CIS chemotherapy.

## 1 Introduction

CIS is widely used as a first-line chemotherapeutic agent against various types of solid tumors (Zhang et al., 2022; Fei et al., 2024). However, dose-dependent nephrotoxicity severely limits its clinical application, with approximately 20%–35% of patients developing acute kidney injury (AKI) following CIS treatment (Thompson and Joy, 2024; Yang et al., 2025). CIS-induced AKI is primarily characterized by proximal renal tubular dysfunction, and no specific and effective therapeutic strategy is currently available in clinical practice (Su et al., 2024). Therefore, the identification of approaches to alleviate CIS-induced nephrotoxicity has become an urgent clinical issue.

The pathogenesis of CIS-induced nephrotoxicity is multifactorial, involving mitochondrial dysfunction, inflammation, oxidative stress, and apoptosis of renal tubular epithelial cells (Tang et al., 2023). Emerging evidence highlights the critical role of metabolic dysregulation in AKI progression. In particular, tryptophan (Trp) metabolism has gained attention as a key regulator of redox balance and renal homeostasis (Wang et al., 2022; Davidson et al., 2022). Trp metabolism proceeds through three principal routes—the kynurenine, serotonin, and indole pathways—which generate a diverse array of bioactive metabolites (Ma et al., 2020; Lee et al., 2021; Zhou et al., 2023). Studies have shown that AKI triggers disturbances in Trp metabolism, and the dysregulated metabolites and metabolic enzymes can further aggravate AKI progression (Dai et al., 2022). For example, the Trp-derived uremic toxin indoxyl sulfate activates the aryl hydrocarbon receptor, triggering reactive oxygen species overproduction and oxidative stress, thereby accelerating renal function deterioration (Yabuuchi et al., 2021). Consequently, interventions targeting Trp metabolism have been recognized as a promising therapeutic approach for AKI, including CIS-induced nephrotoxicity.

In recent years, ferroptosis—an iron-dependent form of regulated cell death driven by glutathione (GSH) depletion and lipid peroxidation—has gained recognition as a key contributor to AKI (Tonnus et al., 2021; Li et al., 2023). The ferroptosis pathway is governed by a delicate balance between pro-ferroptotic and anti-ferroptotic factors (Yang et al., 2024). Glutathione peroxidase 4 (GPX4) serves as a central defender against lipid peroxidation, and its activity is sustained by the cystine/glutamate antiporter xCT, which supplies cysteine for GSH biosynthesis (Deng et al., 2023; Hu et al., 2022; Hui et al., 2026). Conversely, acyl-CoA synthetase long-chain family member 4 (ACSL4) facilitates ferroptosis by incorporating oxidizable polyunsaturated fatty acids into cellular membranes (Poindessous et al., 2024). Dysregulation of this xCT/GPX4/ACSL4 axis has been implicated in CIS-induced renal damage, making ferroptosis an attractive therapeutic target for AKI.

ART, a semi-synthetic derivative of artemisinin with favorable water solubility, is widely used as a first-line antimalarial agent with an established safety profile (Dai et al., 2025). Beyond its antimalarial activity, drug repurposing studies have revealed that ART exhibits numerous bioactivities, including anti-inflammatory, antiviral, and anti-tumor effects (Chang et al., 2025; Hu et al., 2025; Ma et al., 2024). Notably, recent studies have demonstrated ART’s protective effects in various organ injury models, including liver and lung injury (Zhang et al., 2026a; Ji et al., 2023). In AKI models, ART protects against sepsis-induced renal injury by regulating the lactate/AMPK/mTOR pathway and restraining excessive autophagy (Chen et al., 2025). However, although previous studies have confirmed the protective efficacy of ART against CIS-triggered AKI via inhibiting macrophage Mincle-mediated tubular epithelial necrosis (Lei et al., 2021), it remains largely unexplored whether ART alleviates CIS nephrotoxicity through reshaping endogenous metabolic homeostasis, especially the potential crosstalk between Trp metabolic reprogramming and ferroptosis in renal tubular cells.

In the present study, we employed an integrated strategy combining untargeted metabolomics and transcriptomics to systematically investigate the renoprotective effect of ART against CIS-induced AKI and its underlying mechanisms. Renal function biomarkers, histopathological evaluation, and molecular analyses were used to assess ART’s therapeutic efficacy. Multi-omics profiling was performed to identify key metabolic pathways and transcriptional programs modulated by ART, followed by targeted validation experiments both *in vivo* and *in vitro*. By integrating multi-omics discovery with mechanistic validation, this study provides a comprehensive insight into ART’s therapeutic potential and establishes a scientific foundation for its future clinical translation in preventing CIS-induced nephrotoxicity.

## 2 Materials and methods

### 2.1 Chemicals and reagents

CIS (C295225, purity ≥99.5%) and ferrostatin-1 (Fer-1, F129882, purity ≥99%) were purchased from Aladdin Biochemical Technology Co., Ltd. (Shanghai, China). Indoxyl sulfate (IS), kynurenic acid (KA), xanthurenic acid (XA) and 5-hydroxyindole-3-acetic acid (HIAA) were purchased from Sigma-Aldrich (Saint Louis, United States). ART (A800614, purity ≥99%) was obtained from Maclin Biochemical Technology Co., Ltd. (Shanghai, China).

### 2.2 Animal experiments

Male C57BL/6 mice (7–8 weeks old) were purchased from the Laboratory Animal Center of Qinglongshan (certificate no. SYXK 2023-005). Animal care and experimental procedures were approved by the Animal Care and Use Committee of Wannan Medical College (No. WNMC-AWE-2024089). Mice were housed under controlled environmental conditions (12 h light/dark cycle, 24 °C ± 2 °C, 55% ± 5% humidity) with free access to water and standard chow.

To assess the nephroprotective effect of ART against CIS-induced AKI, mice were randomly divided into four groups (n = 6/group): normal control (NC), CIS (15 mg/kg), ART (25 mg/kg) + CIS, and ART (50 mg/kg) + CIS. By referencing published studies (Cao et al., 2016; Zhu et al., 2024) and carrying out preliminary dose screening within the typical administration range, we adopted 25 mg/kg and 50 mg/kg ART to investigate its dose-related renoprotective effects. ART was administered once daily by oral gavage for five consecutive days. On day 5, a single intraperitoneal injection of CIS was given to induce AKI.

To further evaluate the therapeutic potential of ART after CIS exposure, an independent therapeutic intervention cohort was included. In this regimen, mice were first subjected to a single intraperitoneal injection of CIS (15 mg/kg), followed by oral administration of ART (50 mg/kg) at 12, 24, 36, and 48 h after CIS injection. All mice were euthanized 72 h after CIS administration by inhalation of 5% isoflurane. Blood and kidney samples were harvested for further analyses.

### 2.3 Estimation of renal function

Serum creatinine (SCr) and blood urea nitrogen (BUN) levels were quantified using commercial kits (Nanjing Jiancheng Bioengineering Institute, China) following the manufacturer’s protocols. Kidney index was determined by the ratio of kidney weight (mg) to body weight (g), expressed as a percentage.

### 2.4 Renal histopathology and immunohistochemistry

Following fixation in 4% paraformaldehyde, kidney tissues were paraffin-embedded and sectioned to a thickness of 4 μm. Sections were subjected to H&E staining for histopathological assessment. Kidney injury was evaluated by two individuals in a blinded manner and scored based on the degree of damage. Renal tubular injury (dilatation, necrosis, and cast formation) was scored as previously described (Cao et al., 2024). Immunohistochemistry for 4-HNE was performed as described above. Images were captured under a light microscope (Nikon, Japan).

### 2.5 Untargeted metabolomics analysis

Kidney tissue samples (80 mg) from the NC, CIS, and ART (50 mg/kg) + CIS groups were homogenized and subjected to ultrasonic extraction. After centrifugation, the homogenate was resuspended in 300 μL of methanol–water and incubated with shaking at 37 °C for 20 min. Following a second centrifugation (13,000 rpm, 10 min), the resulting supernatant was collected and stored at −80 °C for subsequent UHPLC-MS/MS analysis.

Sample analysis was conducted on a Vanquish UHPLC system coupled to an Orbitrap Q ExactiveTM HF mass spectrometer (Thermo Fisher, Germany). Separation was performed on a Hypersil Gold column (2.1 mm × 100 mm, 1.9 µm) maintained at 4 °C, using a mobile phase of 0.1% formic acid in water (A) and methanol (B) at 0.2 mL/min. The gradient program was as follows: 1.5–3 min, 2%–85% B; 3–10 min, 85%–100% B; 10–10.1 min, 100%–2% B; 10.1–12 min, 2% B. Mass spectra were acquired in positive and negative polarity modes with key parameters: spray voltage, 3.5 kV; capillary temperature, 320 °C; auxiliary gas heater temperature, 350 °C; sheath gas, 35 psi; auxiliary gas, 10 L/min.

Raw data were processed using XCMS software, which included filtering by retention time and m/z ratio, peak alignment, and peak extraction based on ppm and adduct information. Metabolite identification was performed by matching MS/MS spectra against the m/z Cloud, m/z Vault, and Mass List databases. Group separation was visualized using principal component analysis (PCA) and partial least squares discrimination analysis (PLS-DA), with model validity assessed by 200-permutation tests. Differential metabolites were defined by VIP > 1, P < 0.05, and fold change (FC) > 1.2 or FC < 0.883. KEGG pathway enrichment analysis was performed using MetaboAnalyst.

### 2.6 Transcriptomic analysis

Kidney tissue samples (100 mg) were collected from the same anatomical location of three mice in the NC, CIS, or ART (50 mg/kg) + CIS groups. Total RNA was extracted, and its concentration and integrity were evaluated using a Nanodrop 2000 and an Agilent 2100 Bioanalyzer. Sequencing libraries were constructed following a standard protocol: mRNA enrichment with Oligo (dT) beads, fragmentation, cDNA synthesis, end repair, A-tailing, adapter ligation, and PCR amplification. After validation of concentration and insert size, paired-end sequencing was conducted on an Illumina NovaSeq platform.

For data processing, quality control, genome alignment, and transcriptome reconstruction were performed using fastp (v0.23.2), HISAT2 (v2.2.1), and StringTie (v2.2.0), respectively. For all samples, sequencing quality and yields were high, with yields from 42 to 47 million reads and Q30 base percentages exceeding 96%. The percentage of trimmed reads aligning to the reference was high, exceeding 92% for all samples, and uniquely mapped reads were also high, with percentages above 75%. In addition, DESeq2 identified differentially expressed genes based on |log2FC| ≥1 and FDR < 0.05. clusterProfiler (v4.6.2) was employed for Gene Ontology (GO) and KEGG pathway enrichment analysis.

### 2.7 Quantification of tryptophan-pathway metabolites

IS, XA, KA, and HIAA were determined using an Agilent 1290 HPLC system coupled to a G6460 triple quadrupole mass spectrometer (Agilent Technologies, Santa Clara, CA, United States). Chromatographic separation was carried out on a Welch Boltimate C18 column (50 × 2.1 mm i.d., 2.7 μm) and a guard column (5 × 2.1 mm i.d., 2.7 μm) with the column temperature maintained at 30 °C. The mobile phase consisted of water containing 0.1% formic acid (mobile phase A) and Acetonitrile (mobile phase B) at a flow of 0.35 mL/min. The gradient elution program started at 2% B, increased linearly to 80% B over 1.6 min, and held for 2.1 min. Subsequently, the mobile-phase composition was linearly reduced back to 2%B within 1 min, and kept at 2% B until the end of the 6-min run.

The electrospray ionization source was performed in both negative and positive ionization modes. The optimal parameters for mass spectrometry conditions were: capillary voltage at 4000 V; drying gas (N2) with a flow rate of 10 L/min and a temperature of 325 °C; delta EMV (+) at 200 V; collision gas (N2); and a dwell time of 200 ms. Optimized precursor-to-product ion transitions for IS, XA, KA, and HIAA are summarized in Supplementary Table S1. The collected and analyzed data were used with the MassHunter B. 06.00 software package.

For sample preparation, 25 μL of kidney homogenate (100 mg kidney tissue per mL saline) and 25 μL of internal standard were mixed with 100 μL acetonitrile. After vortexing and centrifugation at 14,000 rpm for 10 min, the supernatants were diluted with the initial mobile phase and subjected to LC-MS/MS measurement of IS, XA, KA, and HIAA.

### 2.8 Cell culture and treatment

A human proximal tubule epithelial cell line (HK-2), human hepatocellular carcinoma cells (Huh 7), and human non-small cell lung cancer cells (A549) were obtained from the Cell Bank of the Chinese Academy of Sciences (Shanghai, China). HK-2 cells were cultured in RPMI-1640 medium with 10% fetal bovine serum, 100 μg/mL streptomycin, and 100 U/mL penicillin at 37 °C under 5% CO_2_. Huh 7 and A549 cells were cultured in DMEM medium.

For cytotoxicity assessment, cells were treated with ART (0, 0.5, 1, 2, 4, 8, 16, 32, 64, or 128 μM) for 24 h. For protection experiments, cells were pretreated with ART (4, 8, or 16 μM) or Fer-1 (0.4 μM) for 24 h, then coincubated with CIS (35 μM) for 24 h. For IS rescue experiments, cells were divided into four groups: control (CON), CIS, CIS + ART, and CIS + ART + IS. After ART pretreatment, HK-2 cells were exposed to CIS with or without 100 μM IS co-treatment for 24 h. To evaluate the influence of ART on CIS anticancer activity, A549 and Huh-7 cells were treated with CIS alone or combined with ART. Cell viability was measured using the CCK-8 assay (KeyGEN, China) according to the manufacturer’s instructions.

### 2.9 Immunofluorescence staining

After fixation in 4% paraformaldehyde and blocking with 10% BSA, HK-2 cells grown on coverslips were sequentially incubated with primary antibodies (overnight, 4 °C) and fluorophore-conjugated secondary antibodies (1 h, 37 °C). Nuclei counterstaining was performed with DAPI, and images were examined under a fluorescence microscope (Nikon, Japan).

### 2.10 Reactive oxygen species (ROS) detection

Intracellular ROS production in HK-2 cells was detected using the DCFH-DA probe (KeyGEN, China). Following incubation with 10 µM DCFH-DA at 37 °C for 20 min, cells were washed, and fluorescence intensity was measured by flow cytometry or fluorescence microscopy.

### 2.11 Glutathione (GSH), malondialdehyde (MDA), and Fe^2+^ assays

After homogenization of kidney tissues and HK-2 cells in cold PBS, GSH and MDA levels were measured with commercial kits (Nanjing Jiancheng Bioengineering Institute, China) following the manufacturer’s protocols. Fe^2+^ levels were measured using a commercial kit from Leagene Biotechnology Co., Ltd. (Beijing, China).

### 2.12 Mitochondrial membrane potential assay

JC-1 staining (Beyotime, China) was performed to detect mitochondrial membrane potential in HK-2 cells following the manufacturer’s protocol. Cells were loaded with JC-1 solution for 20 min at 37 °C, washed, and observed and photographed using a confocal laser scanning microscope (Li et al., 2024).

### 2.13 Transmission electron microscopy

For transmission electron-microscopy assessment of mitochondrial morphology, kidney tissues were harvested and fixed in 1.25% glutaraldehyde prepared in 0.1 M phosphate buffer, followed by post-fixation in 1% OsO_4_ diluted with 0.1 M phosphate buffer. Tissues were then ultrathin-sectioned (60 nm thickness) using an ultramicrotome, mounted onto copper grids, and stained with uranyl acetate and lead citrate. Sections were examined under a transmission electron microscope. Five random visual fields from blinded samples were imaged.

### 2.14 Oxidized lipids measurement

Lipid peroxidation was assessed using the C11-BODIPY 581/591 fluorescent probe. HK-2 cells were cultured in 24-well plates until reaching 80% confluence, with three biological replicate wells per condition. Cells were incubated with C11-BODIPY 581/591 probe (10 μM) at 37 °C for 25 min. After washing with pre-warmed PBS, cellular fluorescence signals were acquired using a confocal microscope.

### 2.15 RT-qPCR

RNA was extracted from kidney tissue or HK-2 cells with TRIzol (TaKaRa, Japan), and its purity and concentration were determined on a NanoDrop 2000 (Thermo Fisher, United States). cDNA was synthesized using the PrimeScript RT Reagent Kit (TaKaRa, Japan). RT-qPCR was carried out in a 10 μL volume containing 1 μL cDNA, 4 μL RNase/DNase-free water, 4 μL SYBR Premix Ex Taq, and specific primers (Supplementary Table S2). Target gene expression was normalized to GAPDH or β-actin and quantified using the 2^−ΔΔCT^ method.

### 2.16 Western blotting

Kidney tissues and HK-2 cells were lysed in RIPA buffer containing PMSF (Beyotime, China). Lysates were clarified by centrifugation, and a BCA protein assay kit was used to quantify protein concentrations. Sample was resolved by 10% SDS-PAGE, electrotransferred onto PVDF membrane (Millipore, United States), and blocked with 5% skim milk at room temperature for 1 h. Membranes were subsequently incubated overnight at 4 °C with primary antibodies against the following proteins: KIM-1 (1:1,000, 30948-1-AP, Proteintech), xCT (1:1,000, A2413, ABclonal), ACSL4 (1:2,000, 22401-1-AP, Proteintech), GPX4 (1:1,000, DF6701, Affinity), FTL (1:2000, 10727-1-AP, Proteintech), β-actin (1:10,000, AC004, ABclonal), and GAPDH (1:5,000, AC002, ABclonal). Membranes were washed and then incubated with secondary antibodies for 1 h at room temperature. Protein bands were visualized via enhanced chemiluminescence, and quantified with ImageJ software. GAPDH or β-actin served as loading controls for normalization.

### 2.17 Data analysis

Results were presented as mean ± SD. Normality of data distribution and homogeneity of variances were assessed before statistical testing. Group differences were analyzed by one-way ANOVA followed by Tukey’s *post hoc* test using SPSS (version 18.0, Chicago, IL). A value of *P* < 0.05 was considered statistically significant.

## 3 Results

### 3.1 ART ameliorates CIS-induced nephrotoxicity in mice

To assess whether ART protects against CIS-induced nephrotoxicity *in vivo*, mice were treated with ART (25 or 50 mg/kg, i.g.) for five consecutive days, with a single CIS injection (15 mg/kg, i.p.) administered on day 5 (Figure 1A). Serum and kidney tissue samples were collected 72 h after CIS injection. CIS administration significantly increased the kidney index, BUN, and SCr, indicative of acute renal dysfunction. ART pretreatment dose-dependently reversed these elevations (*P* < 0.05; Figures 1B–D).

**FIGURE 1:**
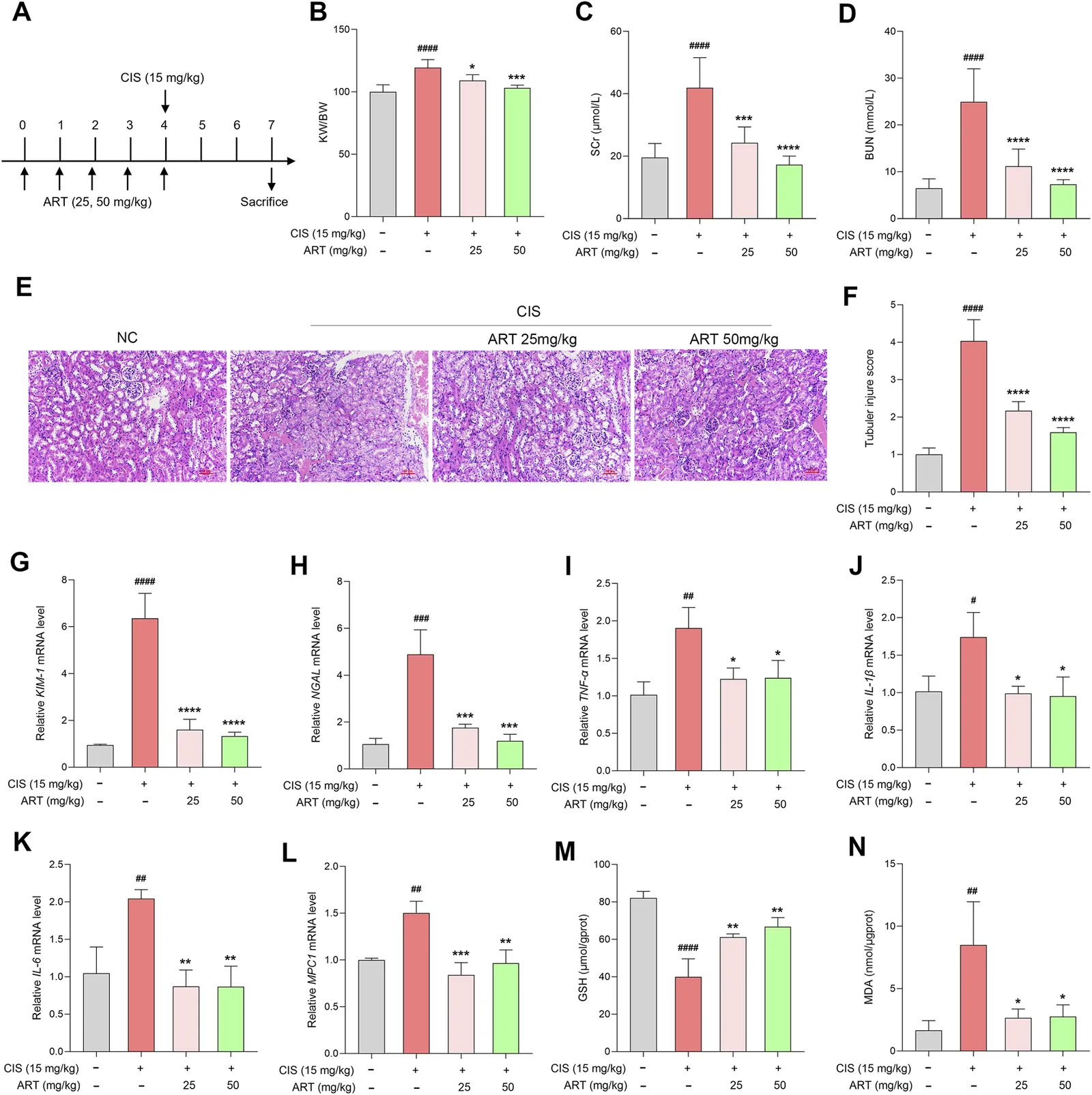
ART ameliorated renal injury in a mouse model of CIS-induced nephrotoxicity. **(A)** Schematic diagram illustrating the establishment of the CIS-induced AKI mouse model. **(B)** Kidney weight/body weight ratio. **(C)** Serum SCr levels. **(D)** Serum BUN levels. **(E)** Representative H&E-stained histopathological images of mouse kidneys (magnification ×400, scale bar: 50 μm). **(F)** Renal injury score. **(G,H)** mRNA expression levels of KIM-1 and NGAL. **(I–L)** mRNA expression levels of TNF-α, IL-1β, IL-6, and MCP1. **(M,N)** Renal GSH and MDA contents. NC, normal control group; CIS, cisplatin group; ART, cisplatin + artesunate group. Data are presented as mean ± SD. (n = 6 mice of each group) ^#^ *P* < 0.05, ^##^ *P* < 0.01, ^###^ *P* < 0.001, ^####^ *P* < 0.0001 vs. NC; *P < 0.05, **P < 0.01, ***P < 0.001, ****P < 0.0001 vs. CIS.

Similarly, renal H&E staining showed that CIS induced significant tubule injury, characterized by tubule dilation, cell necrosis, and protein cast formation. Notably, ART pretreatment markedly ameliorated these histopathological lesions (Figures 1E,F). Furthermore, ART pretreatment dose-dependently downregulated the mRNA expression of NGAL and KIM-1, two well-recognized markers of renal tubular damage (*P* < 0.05; Figures 1G,H). These data establish that ART effectively protects against CIS-induced nephrotoxicity *in vivo*.

To further explore the therapeutic potential of ART against CIS-provoked renal injury, we delivered 50 mg/kg ART via intragastric gavage at 12, 24, 36, and 48 h following CIS injection (Supplementary Figure S1A). Measurements of kidney index, serum BUN, and SCr collectively confirmed that post-administration of ART markedly alleviated CIS-triggered nephrotoxicity (Supplementary Figures S1B–D).

### 3.2 ART suppresses CIS-induced renal inflammation and oxidative stress

Given the pivotal roles of inflammation and oxidative stress in CIS-induced AKI, we measured these parameters in renal tissues. RT-qPCR was used to determine the mRNA levels of key pro-inflammatory mediators, including tumor necrosis factor-α (TNF-α), interleukin-1β (IL-1β), interleukin-6 (IL-6), and monocyte chemoattractant protein-1 (MCP1). The expression of these cytokines was significantly elevated in the kidneys of CIS-treated mice compared to the NC group. Notably, ART pretreatment markedly suppressed this elevation (Figures 1I–L).

Moreover, CIS exposure disrupted renal redox balance, characterized by depleted GSH activity and elevated MDA levels—both of which were normalized by ART administration (Figures 1M,N). These findings indicate that ART’s renoprotective effect is accompanied by potent anti-inflammatory and anti-oxidative effects.

### 3.3 ART restores metabolic profiles in CIS-induced nephrotoxicity

To investigate the metabolic basis of ART’s renoprotective effects, we performed untargeted metabolomic analysis on kidney tissues from NC, CIS, and ART-treated mice. Principal component analysis (PCA) showed distinct clustering of the three groups, reflecting substantial metabolic reprogramming induced by CIS that was markedly reversed by ART (Figures 2A,B). Hierarchical clustering heatmaps revealed that the metabolic perturbation caused by CIS was markedly attenuated by ART treatment (Figures 2C,D).

**FIGURE 2:**
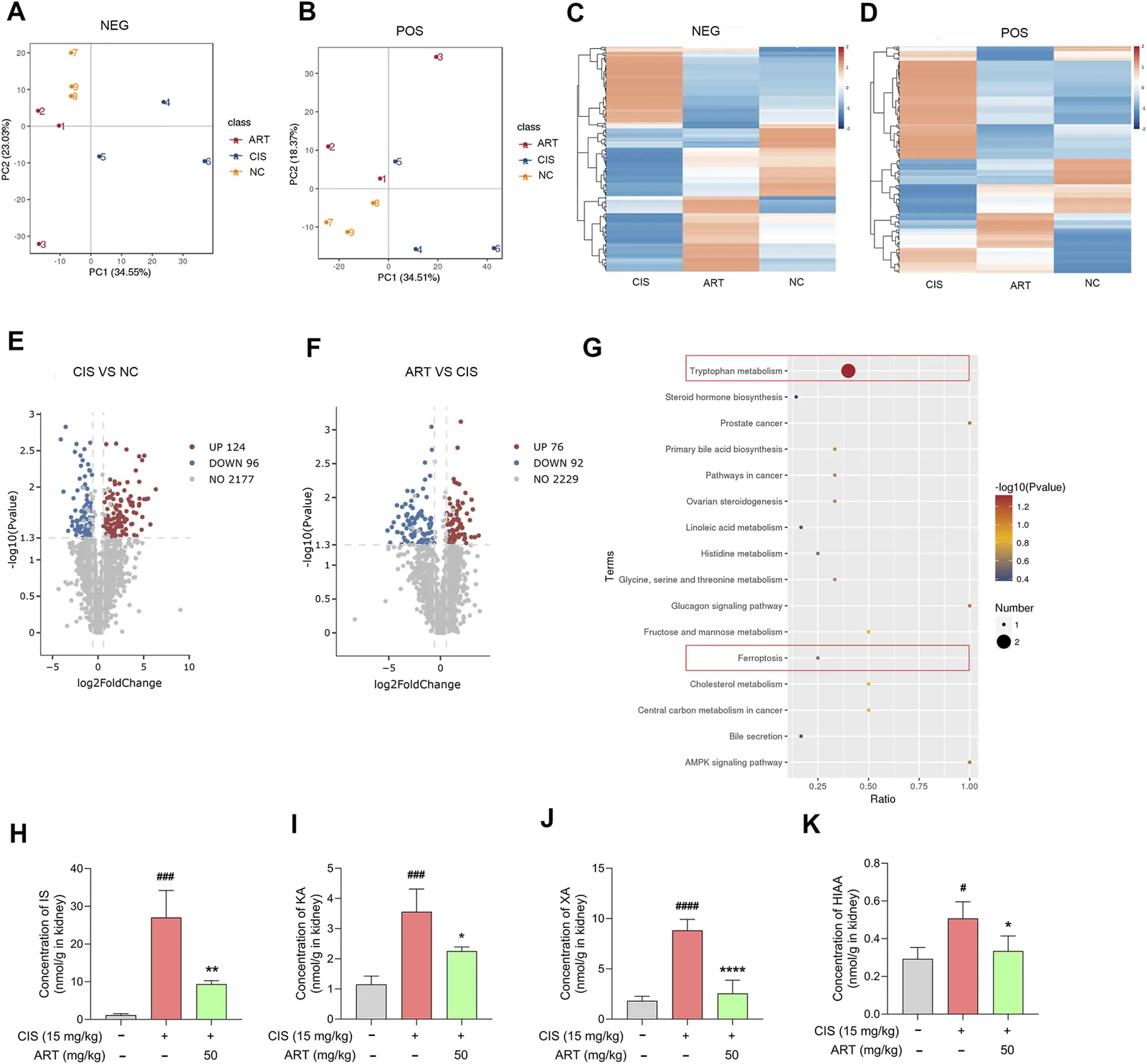
ART restored the metabolic disturbance in CIS-challenged kidney tissues. **(A,B)** PCA scatter plots of kidney samples from the NC, CIS, and ART groups. **(C,D)** Heat maps of differentially abundant metabolites in kidney samples detected in negative and positive ion modes. **(E,F)** Volcano plots of differentially abundant metabolites in mouse kidney samples. **(G)** KEGG pathway enrichment analysis of differentially abundant metabolites in mouse kidney samples from ART vs. CIS groups. **(H–K)** Absolute quantitative detection of tryptophan metabolic metabolites: **(H)** IS, **(I)** KA, **(J)** XA, **(K)** HIAA. Data are presented as mean ± SD.^#^ *P* < 0.05, ^###^ *P* < 0.001, ^####^ *P* < 0.0001 vs. NC; *P < 0.05, **P < 0.01, ****P < 0.0001 vs. CIS (n = 3 mice for untargeted metabolomics; n = 6 mice for absolute metabolite quantification). NC, normal control group; CIS, cisplatin group; ART, cisplatin + artesunate group; IS, indoxyl sulfate; KA, kynurenic acid; XA, xanthurenic acid; HIAA, 5-hydroxyindole-3-acetic acid.

Volcano plot analysis revealed 124 upregulated and 96 downregulated metabolites in the CIS group relative to the NC group (Figure 2E). After ART treatment, 76 metabolites were upregulated and 92 metabolites were downregulated (Figure 2F). KEGG pathway analysis of differentially abundant metabolites pinpointed Trp metabolism and ferroptosis as significantly dysregulated pathway following ART treatment (Figure 2G). Specifically, untargeted metabolomics revealed that key metabolites in the kynurenine pathway (e.g., xanthurenic acid) and indole pathway (e.g., indoxyl sulfate) were elevated in CIS-treated kidneys. Further targeted quantitative verification was conducted on indoxyl sulfate, kynurenine acid, xanthurenic acid, and 5-hydroxyindole-3-acetic acid. Quantitative results confirmed that CIS induced significant accumulation of these metabolites, whereas ART pretreatment restored their levels (Figures 2H–K).

### 3.4 Renal transcriptomic analysis of ART’s renoprotective effects

To complement the metabolic findings and gain deeper mechanistic insight, we performed RNA sequencing on kidney tissues from the three experimental groups. PCA of the transcriptomic data revealed three distinct clusters corresponding to NC, CIS, and ART-treated groups, confirming that ART substantially reversed CIS-induced transcriptional reprogramming (Figure 3A). Hierarchical clustering heatmaps revealed that the transcriptomic perturbation caused by CIS was markedly attenuated by ART treatment (Figure 3B). Volcano plot analysis identified 2105 upregulated and 1805 downregulated genes in CIS-treated kidneys versus controls (Figure 3C). After ART treatment, these numbers shifted to 1,304 genes upregulated and 1,545 downregulated genes (Figure 3D).

**FIGURE 3:**
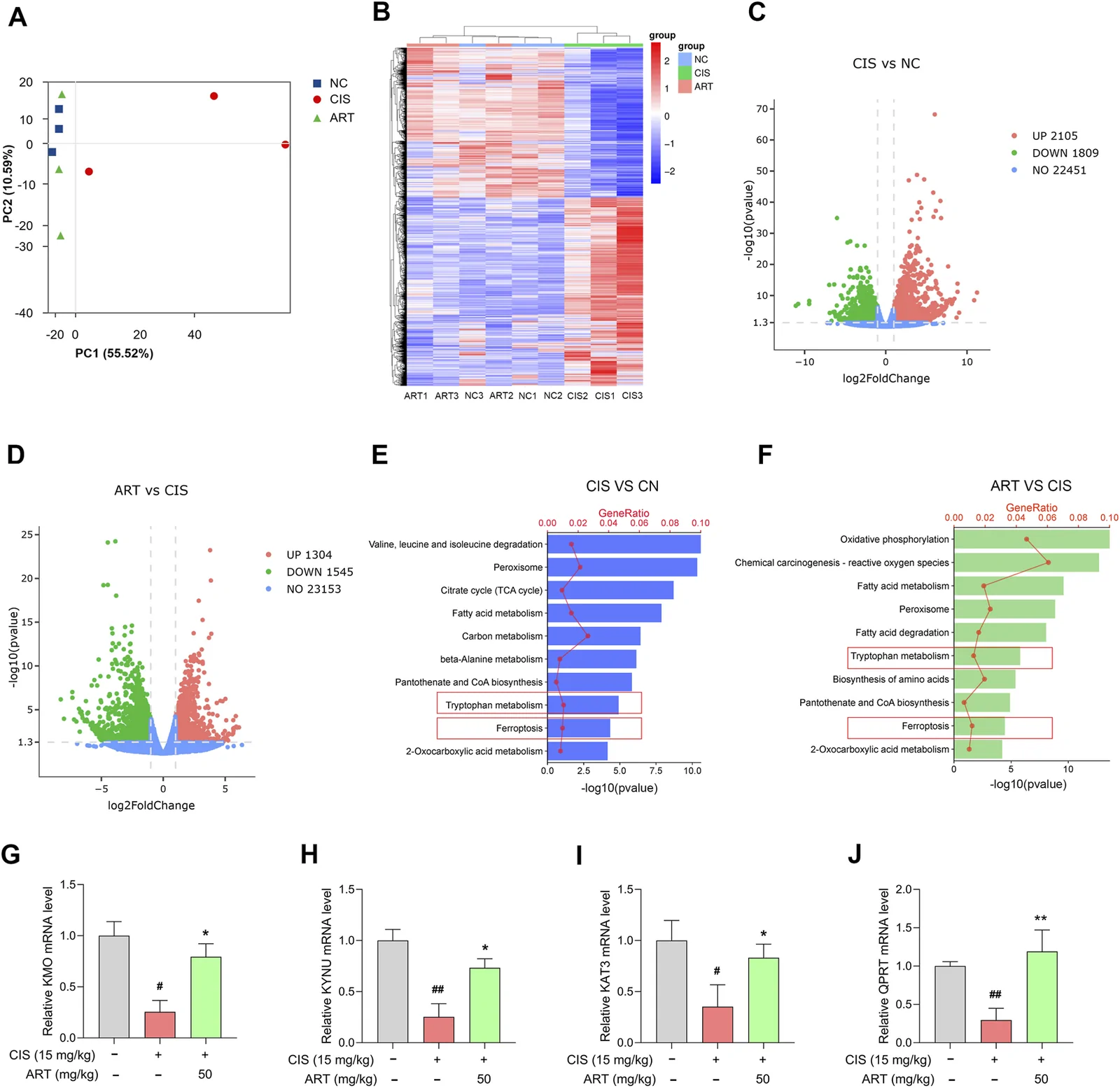
Transcriptomic analysis of ART-treated AKI mice. **(A)** PCA score plots of renal transcriptomes from the NC, CIS, and ART groups. **(B)** Heat maps of differentially abundant genes in kidney samples from the NC, CIS, and ART groups. **(C,D)** Volcano plots showing differentially expressed genes CIS vs. NC **(C)** and ART vs. CIS **(D)** (|log2 fold change| ≥ 1; *P* < 0.05). **(E,F)** KEGG pathway enrichment analysis of differentially expressed genes in CIS vs. NC **(E)** and ART vs. CIS **(F)**. **(G–J)** mRNA expression levels of KMO, KYNU, KAT3 and QPRT. Data are presented as mean ± SD. ^#^ *P* < 0.05, ^##^ *P* < 0.01 vs. NC; *P < 0.05, **P < 0.01 vs. CIS. (n = 3 mice for transcriptomic analysis; n = 6 for qPCR-based mRNA expression analysis) NC, normal control group; CIS, cisplatin group; ART, cisplatin + artesunate group.

KEGG enrichment analysis identified Trp metabolism and ferroptosis as significantly dysregulated pathways in CIS-injured kidneys, both of which were notably modulated by ART treatment (Figures 3E,F). To further verify the transcriptomic results, RT-qPCR was used to quantify the mRNA levels of tryptophan metabolic genes KMO, KYNU, KAT3 and QPRT. In line with transcriptome data, CIS significantly inhibited the expression of these genes, and ART pretreatment reversed this inhibitory effect (Figures 3G–J). Collectively, transcriptomic analysis confirms that modulation of Trp metabolism and ferroptosis constitutes the core mechanism by which ART alleviates CIS-induced nephrotoxicity.

### 3.5 ART inhibits CIS-induced nephrotoxicity in mice by suppressing ferroptosis

To elucidate the mechanism by which ART inhibits CIS-induced nephrotoxicity, we systematically investigated its effects on ferroptosis. Compared to controls, the CIS group displayed a marked elevation in Fe^2+^ levels, which was significantly reversed by ART treatment (Figure 4A). Notably, CIS challenge significantly upregulated the mRNA expression of the pro-ferroptotic genes ACSL4 and PTGS2, whereas ART treatment markedly downregulated their expression (*P* < 0.05) (Figures 4B,C). Additionally, CIS suppressed the mRNA expression of the ferroptosis defender GPX4, an effect that was effectively restored by ART treatment (*P* < 0.05) (Figure 4D).

**FIGURE 4:**
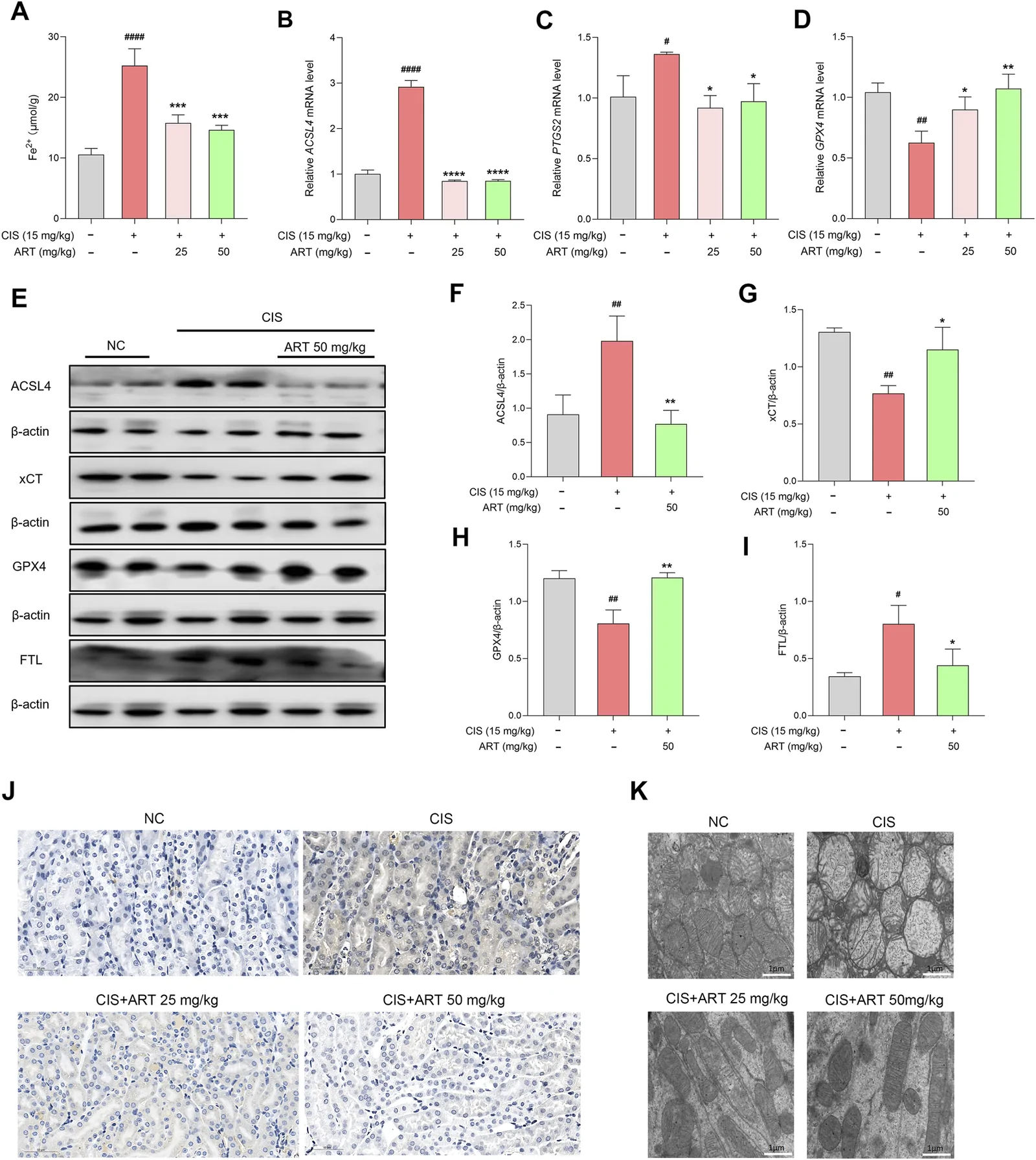
ART inhibits CIS-induced ferroptosis in AKI mice. **(A)** Fe^2+^ levels in kidney tissues. **(B–D)** mRNA expression levels of ACSL4, PTGS2, and GPX4. **(E)** Representative western blots of ACSL4, xCT, GPX4 and FTL protein expression. **(F–I)** Densitometric quantification of ACSL4, xCT, GPX4 and FTL protein levels. **(J)** Representative immunohistochemical staining of 4-HNE. **(K)** Transmission electron microscopy showing mitochondrial ultrastructure in mouse renal tissue. NC, normal control group; CIS, cisplatin group; ART, cisplatin + artesunate group. Data are presented as mean ± SD (n = 6 mice of each group). ^#^ *P* < 0.05, ^##^ *P* < 0.01, ^###^ *P* < 0.001, ^####^ *P* < 0.0001 vs. NC; *P < 0.05, **P < 0.01, ***P < 0.001, ****P < 0.0001 vs. CIS.

Moreover, western blot analysis showed that CIS challenge significantly increased ACSL4 and FTL protein expression, an effect that was markedly reversed by ART treatment (*P* < 0.05) (Figures 4E–G). Additionally, CIS exposure resulted in a pronounced decrease in GPX4 and xCT protein levels, both of which were effectively restored by ART administration (*P* < 0.05) (Figures 4H,I).

Immunohistochemical staining revealed robust accumulation of 4-HNE, a marker of lipid peroxidation, in renal tissues from CIS-treated mice, and this elevation was largely abrogated following ART intervention (Figure 4J). Consistently, typical ferroptosis-associated mitochondrial ultrastructural lesions triggered by CIS were substantially alleviated upon ART treatment (Figure 4K). These findings collectively demonstrate that ART inhibits CIS-induced nephrotoxicity by modulating ferroptosis pathways.

### 3.6 ART relieves CIS-induced damage in HK-2 cells

Given the proximal tubular epithelial cells are the primary target of CIS-induced nephrotoxicity, we employed the human HK-2 cell line to evaluate the protective effects of ART *in vitro*. The results showed that ART concentrations up to 32 μM were safe, while significant inhibition of cell viability was observed at concentrations exceeding 64 μM (Figure 5A). To evaluate the cytoprotective effect of ART, HK-2 cells were pretreated with ART at 4, 8, or 16 μM, or with Fer-1 (a ferroptosis inhibitor, used as a positive control) prior to co-incubation with 35 μM CIS. CCK-8 assay results showed that ART reversed CIS-induced cytotoxicity in a dose-dependent manner, with the protective effect at 16 μM comparable to that of Fer-1 (Figure 5B).

**FIGURE 5:**
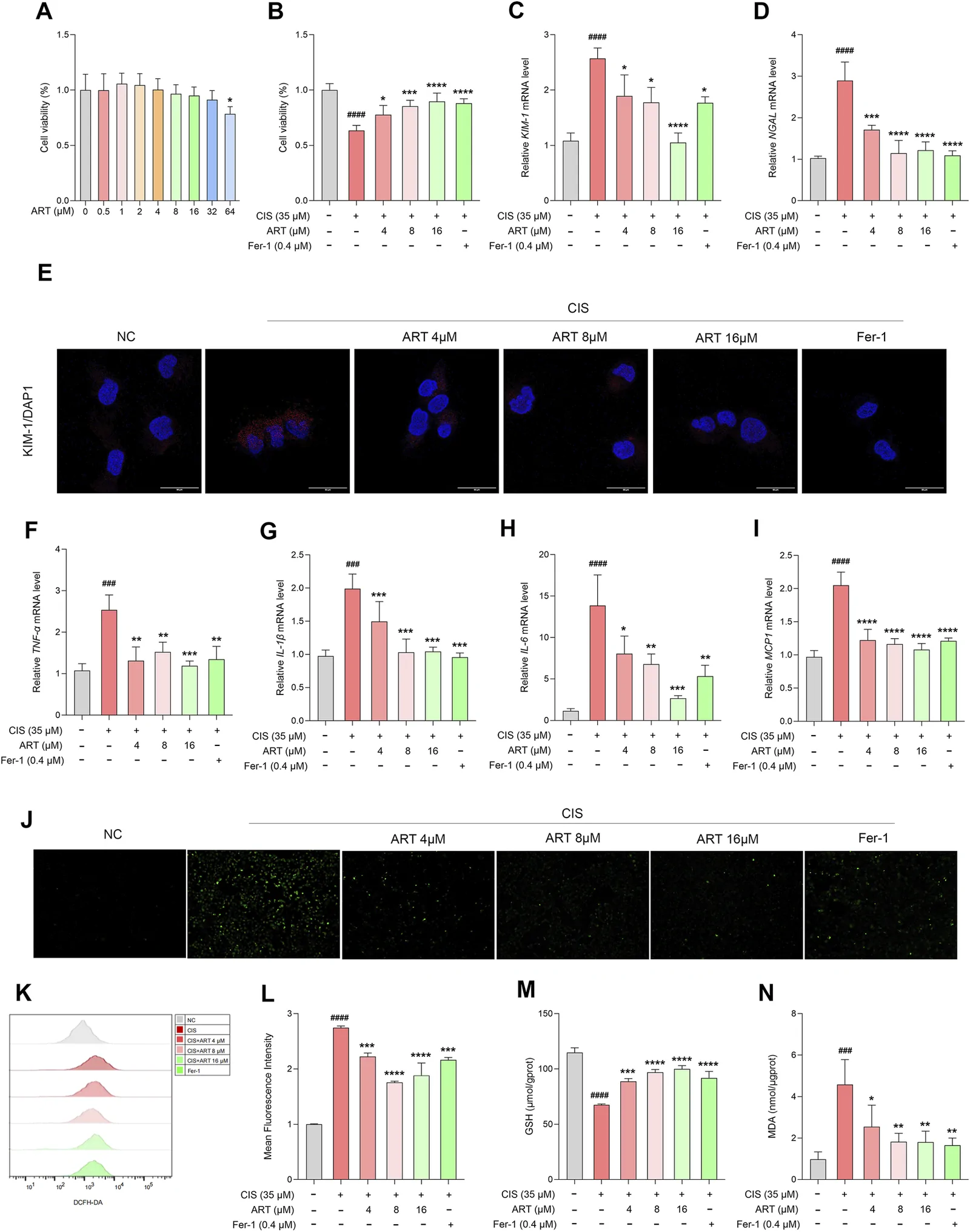
ART protects HK-2 cells against CIS-induced injury. **(A)** Effect of ART on HK-2 cell viability at different concentrations. **(B)** ART restored cell viability after CIS exposure. **(C,D)** mRNA expression levels of KIM-1 and NGAL. **(E)** Representative immunofluorescence images of KIM-1 expression. **(F–I)** mRNA expression levels of TNF-α, IL-1β, IL-6, and MCP1. **(J)** Representative images of DCFH-DA staining in HK-2 cells. **(K,L)** Cellular ROS levels measured using flow cytometry. **(M,N)** GSH and MDA levels in HK-2 cells. NC, normal control group; CIS, cisplatin group; ART, cisplatin + artesunate group. Data are presented as mean ± SD. ^###^ *P* < 0.001, ^####^ *P* < 0.0001 vs. NC; *P < 0.05, **P < 0.01, ***P < 0.001, ****P < 0.0001 vs. CIS.

To further validate the protective effect of ART, we examined its impact on the tubular injury markers NGAL and KIM-1. RT‒qPCR analysis showed that CIS markedly upregulated both markers in HK-2 cells, whereas ART alleviated CIS-induced damage in a dose-dependent manner, with its optimal renoprotective effect comparable to that of Fer-1 (Figures 5C,D). Consistent with these findings, immunofluorescence staining confirmed that ART pretreatment suppressed CIS-induced KIM-1 expression (Figure 5E).

### 3.7 ART suppresses inflammation, oxidative stress and ferroptosis in CIS-exposed HK-2 cells

RT-qPCR analysis revealed that ART markedly reduced the mRNA levels of TNF-α, IL-1β, IL-6, and MCP1 in CIS-treated cells (Figures 5F–I). Flow cytometry using the DCFH-DA probe showed that the relative ROS level was significantly higher in HK-2 cells after 24 h of CIS incubation compared with the NC group, whereas ART treatment inhibited intracellular ROS accumulation induced by CIS (Figures 5J–L). In addition, CIS exposure significantly decreased GSH levels and increased MDA levels, while ART pretreatment significantly restored these parameters (Figures 5M,N), indicating that ART antagonizes oxidative stress. These findings suggest that ART inhibits CIS-induced inflammatory response and oxidative stress *in vitro*.

We next analyzed the mRNA and protein expression of key ferroptosis regulators. At the transcriptional level, both ART and Fer-1 inhibited the CIS-induced upregulation of ACSL4 and PTGS2 (Figures 6B,C), while restoring the CIS-induced downregulation of GPX4 (Figure 6D). These observations were validated at the protein level. CIS challenge significantly increased ACSL4 and FTL protein expression, whereas ART treatment reduced their levels (*P* < 0.05). Furthermore, CIS suppressed xCT expression, an effect that was normalized by ART treatment (*P* < 0.05) (Figures 6E–H).

**FIGURE 6:**
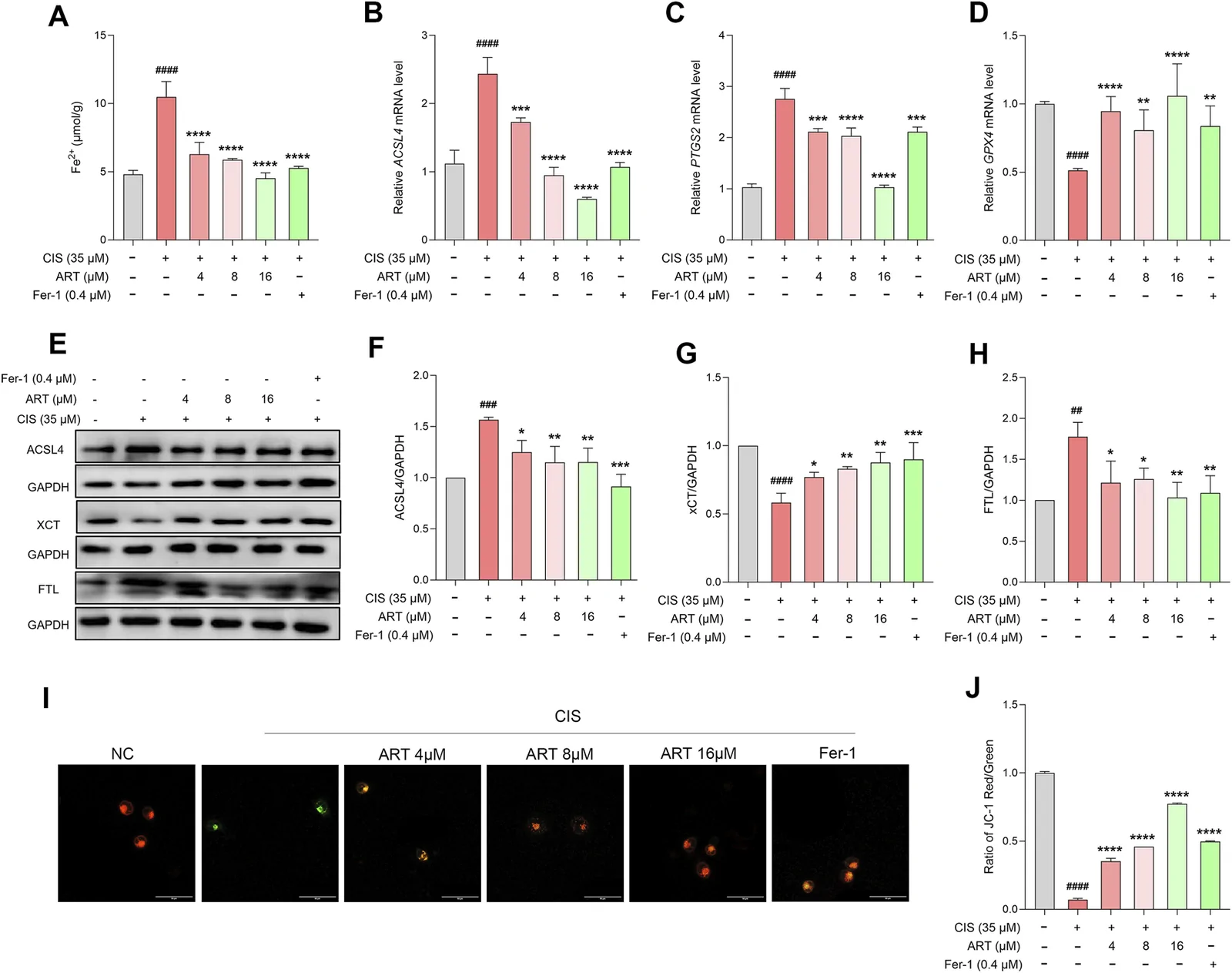
ART inhibits CIS-induced ferroptosis in HK-2 cells. **(A)** Fe^2+^ concentration in HK-2 cells. **(B–D)** mRNA expression levels of ACSL4, PTGS2, and GPX4. **(E)** Representative western blots of ACSL4, xCT, and FTL protein expression. **(F–H)** Densitometric quantification of ACSL4, xCT, and FTL protein levels. **(I,J)** Mitochondrial membrane potential assessed by JC-1 staining in HK-2 cells. NC, normal control group; CIS, cisplatin group; ART, cisplatin + artesunate group. Data are presented as mean ± SD. ^##^ *P* < 0.01, ^###^ *P* < 0.001, ^####^ *P* < 0.0001 vs. NC; *P < 0.05, **P < 0.01, ***P < 0.001, ****P < 0.0001 vs. CIS.

Compared with the NC group, the CIS-treated group displayed a pronounced elevation in intracellular Fe^2+^ levels, which was markedly reversed by ART intervention (Figure 6A). Given that mitochondrial damage is a hallmark of ferroptosis, we next measured mitochondrial membrane potential using the JC-1 probe. ART treatment significantly ameliorated CIS-induced mitochondrial dysfunction, as reflected by restored membrane potential (Figures 6I,J). Together, these findings demonstrate that ART effectively inhibits CIS-induced ferroptosis in HK-2 cells.

### 3.8 IS counteracts the anti-ferroptotic effect of ART in CIS-treated HK-2 cells

First, we evaluated the cytotoxicity of serial concentrations of IS in HK-2 cells. No obvious cell viability loss was observed below 200 μM IS, while 500 μM IS induced significant cell death (Figure 7A). Accordingly, 100 μM IS was selected for subsequent rescue experiments. Cell viability assays showed that CIS markedly reduced HK-2 cell viability. ART co-treatment effectively rescued cell injury induced by CIS, whereas additional IS incubation significantly abrogated this protective effect (Figure 7B). C11-BODIPY 581/591 immunofluorescence staining was applied to detect lipid ROS, a hallmark of ferroptosis. The elevated lipid ROS triggered by CIS was largely suppressed by ART. However, the inhibitory effect of ART on lipid peroxidation was partially reversed upon IS supplementation (Figure 7C).

**FIGURE 7:**
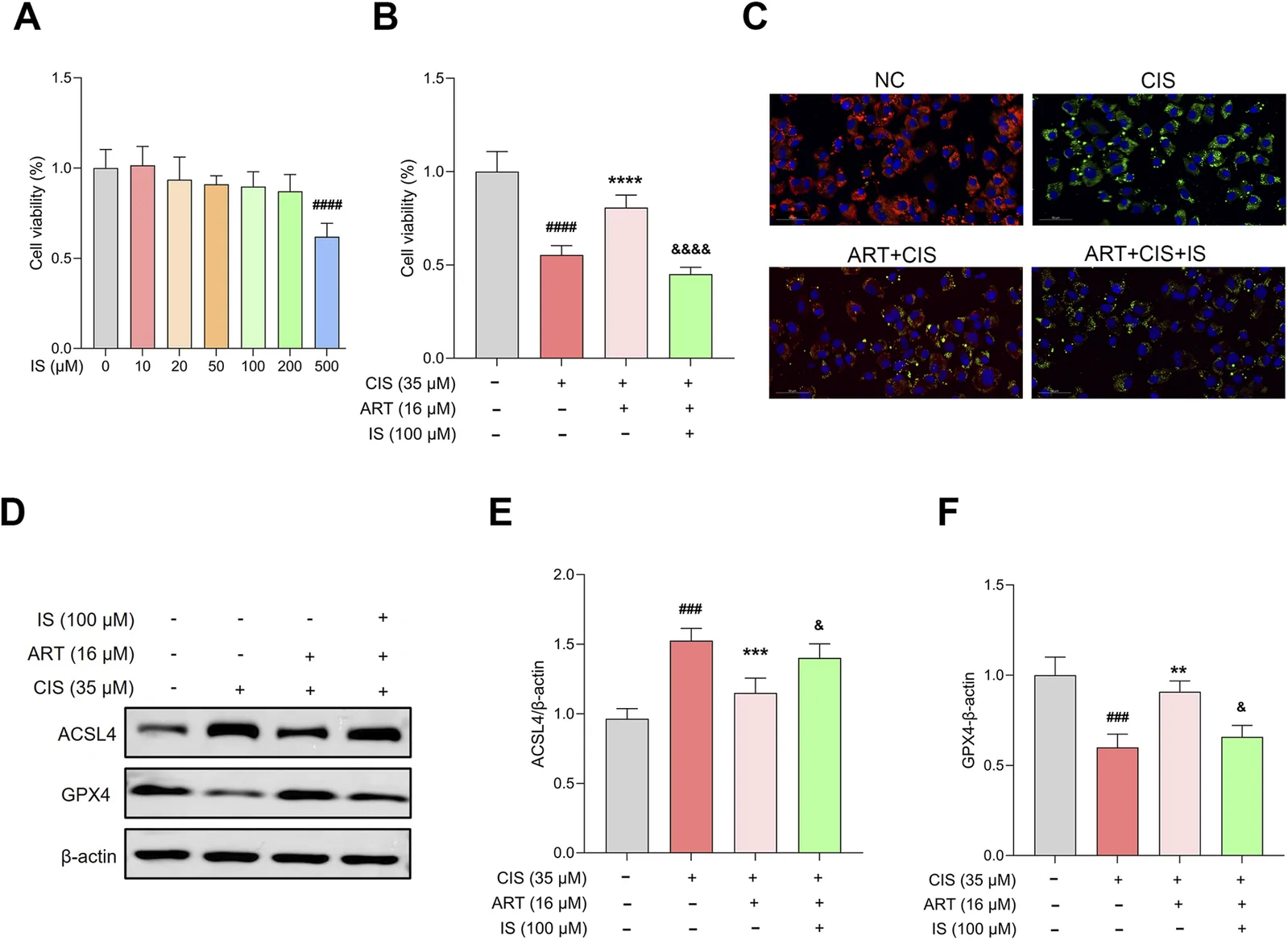
IS counteracts ART-mediated anti-ferroptotic protective effects in CIS-stimulated HK-2 cells. **(A)** Cell viability of HK-2 cells treated with serial concentrations of IS (10–500 μM) for 24 h. **(B)** Cell viability analysis in HK-2 cells treated with CON, CIS, CIS + ART, and CIS + ART + IS groups. **(C)** Representative confocal images of C11-BODIPY 581/591 staining for detection of lipid ROS. Scale bar = 50 μm. **(D)** Western blot bands of ACSL4 and GPX4. β-actin was used as the internal reference. **(E,F)** Densitometric quantification of ACSL4 and GPX4 protein levels. Data are presented as mean ± SD. ^###^ *P* < 0.001, ^####^ *P* < 0.0001 vs. NC; **P < 0.01, ***P < 0.001, ****P < 0.0001 vs. CIS; ^&^ *P* < 0.05, ^&&&&^ *P* < 0.0001 vs. CIS + ART.

Consistent with cellular phenotypic changes, western blot results demonstrated that ART restored GPX4 expression and decreased ACSL4 abundance in CIS-exposed HK-2 cells. Importantly, IS co-treatment offset these regulatory effects of ART: GPX4 levels declined and ACSL4 expression increased again in the CIS + ART + IS group (Figures 7D–F). Taken together, these experiments confirm that the Trp metabolite IS antagonizes ART’s anti-ferroptotic and cytoprotective activity.

## 4 Discussion

CIS is a highly effective first-line chemotherapeutic agent against various solid tumors. However, its clinical application is severely limited by adverse reactions, particularly nephrotoxicity. Currently, no effective preventive strategies are available, making it imperative to explore novel renoprotective agents. ART possesses well-documented anti-inflammatory and anti-oxidative properties. This study systematically evaluated the protective effect of ART against CIS-induced nephrotoxicity both in a mouse model and in HK-2 human renal proximal tubular cells, and further explored the underlying mechanisms. Our results demonstrated that ART significantly alleviates CIS-induced nephrotoxicity by restoring Trp metabolic homeostasis and inhibiting ferroptosis, inflammation, and oxidative stress (Figure 8).

**FIGURE 8:**
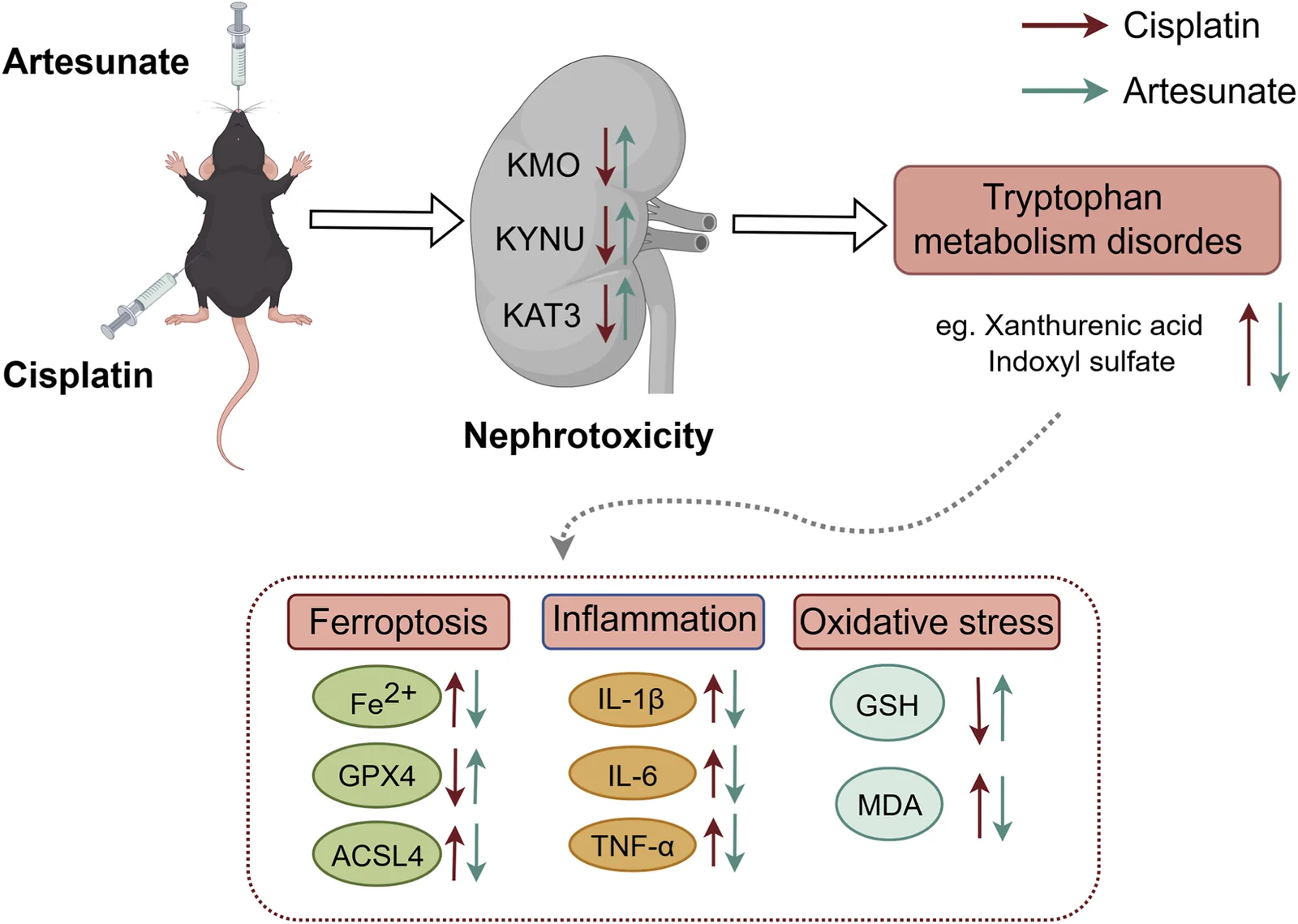
Schematic diagram illustrating potential renoprotective effects of ART against CIS-induced nephrotoxicity. ART treatment is associated with normalization of perturbed Trp metabolism, and mitigation of ferroptosis-related alterations, inflammatory responses, and oxidative stress, which may collectively contribute to renoprotection.

We first demonstrated that ART effectively ameliorates CIS-induced nephrotoxicity, as reflected by reductions in BUN and SCr levels, attenuated histopathological damage, and suppressed expression of KIM-1 and NGAL. These functional improvements were accompanied by marked suppression of inflammatory cytokines (TNF-α, IL-1β, IL-6, MCP1) and restoration of redox balance (GSH, MDA). Importantly, apart from the prophylactic efficacy verified by the pretreatment scheme, ART also exerted obvious renoprotective effects when applied after CIS exposure, indicating ART could be adopted not only for prevention but also for early intervention once renal injury begins. These observations are in line with previous reports of ART’s anti-inflammatory and anti-oxidative properties (Chen et al., 2025; Lei et al., 2021) and extend them to the context of CIS-induced nephrotoxicity, providing a solid phenotypic foundation for subsequent mechanistic exploration.

Recently, multi-omics approaches including metabolomics, proteomics, transcriptomics, and gut-microbiome sequencing have been widely applied to systematically explore pharmacological mechanisms underlying various diseases (Wu et al., 2026; Sharma et al., 2023; Song et al., 2024). Zhang et al. integrated metabolomic, lipidomic, and transcriptomic demonstrated that Erzhi Pills improve renal function, reduce inflammation, and alleviate fibrosis by regulating arachidonic acid metabolism (Zhang et al., 2026b). Our untargeted metabolomic analysis revealed that CIS significantly altered the metabolic profile of renal tissues, with differential metabolites mainly enriched in Trp metabolism and ferroptosis pathways. Strikingly, ART intervention normalized key metabolites in the kynurenine pathway and indole pathway. Complementing these findings, transcriptomic analysis confirmed that ART reversed CIS-induced dysregulation of genes involved in tryptophan metabolism and ferroptosis.

The clinical relevance of Trp metabolism in AKI is well-documented. Elevated levels of kynurenine pathway metabolites, such as kynurenic acid, have been reported in ICU patients with AKI and correlate with poor clinical outcomes (Aregger et al., 2018; Nadour et al., 2022). Mechanistically, metabolites such as xanthurenic acid contribute to proinflammatory cytokine production, including TNF-α and IFN-γ (Umeda et al., 2025). Similarly, CIS-induced AKI is accompanied by disturbed indole pathway metabolism and accumulation of Trp-derived uremic toxins such as indoxyl sulfate, which induce inflammation and oxidative stress (Yabuuchi et al., 2021). In line with these findings, our study showed that ART downregulated xanthurenic acid, indoxyl sulfate, and other metabolites in mice with CIS-induced AKI. These results indicate that ART inhibits CIS-induced nephrotoxicity partly by restoring Trp metabolic homeostasis. Further studies are needed to identify the key Trp metabolites responsible for ART-mediated protection and to clarify their precise regulatory mechanisms.

Mounting evidence has verified that ferroptosis represents a pivotal pathogenic driver of CIS-induced nephrotoxicity. The involvement of ferroptosis in ART’s renoprotective mechanism was further validated through targeted molecular analyses. CIS-challenged kidneys exhibited hallmark features of ferroptosis: elevated Fe^2+^ accumulation, GSH depletion, and lipid peroxidation, which were consistent with previous reports (Bayır et al., 2023). ART intervention effectively reversed these abnormalities. Mechanistically, cellular sensitivity to ferroptosis is closely linked to the expression of iron metabolism proteins, particularly ferritin light chain (FTL), which serves both as a marker of intracellular iron storage and a modulator of ferroptosis susceptibility. We observed that CIS treatment triggered the accumulation of labile free iron, accompanied by compensatory upregulation of total FTL protein. ART treatment reduced FTL expression and alleviated intracellular iron overload. Beyond iron metabolism, ART profoundly modulated the xCT/GPX4/ACSL4 axis, a central regulatory hub of ferroptosis. Specifically, ART restored CIS-induced suppression of the cystine/glutamate antiporter xCT and the lipid repair enzyme GPX4, while downregulating the pro-ferroptotic acyl-CoA synthetase ACSL4. Given that xCT maintains GPX4 activity by supplying cysteine for GSH biosynthesis, and ACSL4 enriches membranes with oxidizable polyunsaturated fatty acids (Poindessous et al., 2024; Abdel-Rahman et al., 2025), our findings suggest that ART exerts dual protective effects: bolstering antioxidant defense while limiting the production of pro-ferroptotic lipid substrates. The functional relevance of these molecular changes was further substantiated in HK-2 cells, where ART demonstrated anti-ferroptotic efficacy comparable to that of the specific inhibitor Fer-1.

Building upon the above results, we further explored the functional connection between Trp metabolism and ferroptosis. Although multi-omics data indicated simultaneous alterations in both pathways after ART treatment, correlative changes cannot confirm their mutual interaction. We therefore performed rescue experiments with IS in HK-2 cells. Exogenous IS partially abrogated ART-mediated cytoprotection against CIS-induced injury. Mechanistically, IS attenuated ART’s inhibitory effect on lipid accumulation and reversed ART-mediated regulation of core ferroptosis mediators GPX4 and ACSL4. These cellular data demonstrate functional crosstalk between Trp metabolism and ferroptosis, and verify that IS can antagonize the anti-ferroptotic activity of ART. Finally, we explored whether ART interferes with CIS anticancer function in A549 and Huh-7 cells. ART co-administration did not reverse CIS-triggered decrease in tumor cell viability (Supplementary Figure S2), suggesting ART does not compromise CIS antitumor activity. This finding strengthens the translational prospect of ART as a protective adjuvant agent to alleviate CIS-induced nephrotoxicity.

Several limitations warrant consideration in this study. First, our multi-omics approach successfully identified the involvement of Trp metabolism and ferroptosis, but it did not pinpoint the precise molecular target of ART. Second, although we verified that exogenous IS counteracts ART’s anti-ferroptotic effect in HK-2 cells, we only tested one Trp metabolite. Further work is required to characterize other altered metabolites and related metabolic enzymes (IDO1, KMO, AhR signaling). In addition, functional validation in animal models will help confirm the interplay between Trp metabolism and ferroptosis *in vivo*. Third, the critical question of whether ART interferes with the anti-tumor efficacy of CIS in a cancer context has only been explored *in vitro* with two cancer cell lines in this work. Given that ART itself possesses anti-cancer properties, future studies in tumor-bearing animal models are essential to evaluate whether ART’s renoprotective effect compromises or potentially enhances CIS’s chemotherapeutic efficacy.

## 5 Conclusion

In this study, our findings suggest that ART confers robust renoprotection against CIS-induced nephrotoxicity. Integrated renal transcriptomic and metabolomic analyses revealed that ART treatment is associated with normalization of perturbed Trp metabolism and suppression of ferroptosis-related molecular alterations in injured kidney tissues. Changes in the xCT/GPX4/ACSL4/FTL axis accompany the protective phenotype upon ART intervention. These molecular changes are coupled with reduced inflammation and oxidative stress and preserved renal tubular function. Collectively, these findings suggest that ART represents a promising therapeutic candidate against CIS-induced nephrotoxicity.

## Data Availability

Raw RNA-sequencing data of this study have been deposited in the NCBI SRA under BioProject accession PRJNA1521679, and BioSample accessions SAMN62808734–SAMN62808742. Metabolomics data files are available from the corresponding author upon reasonable request.
