# Supplementary material for: Artesunate alleviates cisplatin-induced nephrotoxicity by regulating tryptophan metabolism and inhibiting ferroptosis
Source: Front Pharmacol. 2026 Sep 14;17:1884026. doi: 10.3389/fphar.2026.1884026 (PMC13617098; doi:10.3389/fphar.2026.1884026)
Supplement: Supplementary file 1 [file Table1.docx]

**Table S1** Multiple reaction monitoring (MRM) of the transitions of compounds.

| Analyte | Ion mode | Q1 (m/z) | Q2 (m/z) | Fragment (eV) | CE (eV) |
| --- | --- | --- | --- | --- | --- |
| Indoxyl sulfate | - | 212.4 | 80.1 | 90 | 14 |
| Salicylic acid (IS) | - | 137.0 | 93.0 | 90 | 20 |
| Kynurenic acid | + | 190.0 | 144.0 | 90 | 16 |
| 5-hydroxyindole acetic acid | + | 192.0 | 146.0 | 90 | 14 |
| Xanthurenic acid | + | 206.0 | 160.2 | 70 | 16 |
| Antipyrine (IS) | + | 189.1 | 106.1 | 90 | 20 |
