# Supplementary material for: Artesunate alleviates cisplatin-induced nephrotoxicity by regulating tryptophan metabolism and inhibiting ferroptosis
Source: Front Pharmacol. 2026 Sep 14;17:1884026. doi: 10.3389/fphar.2026.1884026 (PMC13617098; doi:10.3389/fphar.2026.1884026)
Supplement: Supplementary file 2 [file Table2.docx]

**Table S2** Forward and reverse primer sequence used for RT-qPCR.

| Target Gene | Forward Primer | Reverse Primer |
| --- | --- | --- |
| Mouse KIM-1 | CAGGGAAGCCGCAGAAAA | GAGACACGGAAGGCAACCAC |
| Mouse NGAL | ACAACCAGTTCGCCATGGTA | AGCTCCTTGGTTCTTCCATACAG |
| Mouse TNF-α | CATCTTCTCAAAATTCGAGTGACAA | TGGGAGTAGACAAGGTACAACCC |
| Mouse MCP1 | TTAAAAACCTGGATCGGAACCAA | GCATTAGCTTCAGATTTACGGGT |
| Mouse IL-1β | GCAACTGTTCCTGAACTCAACT | ATCTTTTGGGGTCCGTCAACT |
| Mouse IL-6 | CTGCAAGAGACTTCCATCCAG | AGTGGTATAGACAGGTCTGTTGG |
| Mouse ACSL4 | TGAACGTATCCCTGGACTAGG | TCAGACAGTGTAAGGGGTGAA |
| Mouse PTGS2 | CACACTCTATCACTGGCACC | TCCAGGAGGATGGAGTTGTT |
| Mouse GPX4 | CATGCCCGATATGCTGAGTGTGG | TAGCACGGCAGGTCCTTCTCTATC |
| Mouse KMO | ATGGCATCGTCTGATACTCAGG | CCCTAGCTTCGTACACATCAACT |
| Mouse KYNU | AGTGGGCTGCACTTTTATACTG | TGCAAACAGGTTGCCTTTCAG |
| Mouse KAT3 | AACCCCGGCGACACCTA | TGATCGTTCTCCTGGTTCCAA |
| Mouse QPRT | CCGGGCCTCAATTTTGCATC | GGTGTTAAGAGCCACCCGTT |
| Mouse GAPDH | AGGTCGGTGTGAACGGATTTG | TGTAGACCATGTAGTTGAGGTCA |
| Human KIM-1 | CTGCAGGGAGCAATAAGGAG | TCCAAAGGCCATCTGAAGAC |
| Human NGAL | GACAACCAATTCCAGGGGAAG | GCATACATCTTTTGCGGGTCT |
| Human TNF-α | AGCCCATGTTGTAGCAAACC | CCTCAGCTTGGATGCTTCTC |
| Human MCP1 | CAGCCAGATGCAATCAATGCC | TGGAATCCTGAACCCACTTCT |
| Human IL-1β | AGCTACGAATCTCCGACCAC | CGTTATCCCATGTGTCGAAGAA |
| Human IL-6 | ACTCACCTCTTCAGAACGAATTG | CCATCTTTGGAAGGTTCAGGTTG |
| Human ACSL4 | TCTGCTTCTGCTGCCCAATT | CGCCTTCTTGCCAGTCTTTT |
| Human PTGS2 | TGCTTGTCTGGAACAACTGC | TGAGCATCTACGGTTTGCTG |
| Human GPX4 | GAGGCAAGACCGAAGTAAACTAC | CCGAACTGGTTACACGGGAA |
| Human β-actin | GGGAAATCGTGCGTGACATT | GGAACCGCTCATTGCCAAT |
